# Rapid Identification of *Vibrio* Species of the Harveyi Clade Using MALDI-TOF MS Profiling With Main Spectral Profile Database Implemented With an In-House Database: Luvibase

**DOI:** 10.3389/fmicb.2020.586536

**Published:** 2020-10-09

**Authors:** Julia Mougin, Christophe Flahaut, Roxane Roquigny, Maryse Bonnin-Jusserand, Thierry Grard, Cédric Le Bris

**Affiliations:** Université du Littoral Côte d’Opale, UMR 1158 BioEcoAgro, Institut Charles Viollette, Unité Sous Contrat ANSES, INRAe, Université d’Artois, Université de Lille, Université de Picardie Jules Verne, Université de Liège, Yncréa, Boulogne-sur-Mer, France

**Keywords:** MALDI-TOF MS, *Vibrio*, Harveyi clade, in-house database, Luvibase, environmental isolates

## Abstract

*Vibrio* bacteria, and particularly members of the Harveyi clade, are the causative agents of vibriosis. This disease is responsible for mass mortality events and important economic losses on aquaculture farms. Improvements in surveillance and diagnosis are needed to successfully manage vibriosis outbreaks. 16S rRNA gene sequencing is generally considered to be the gold standard for bacterial identification but the cost and long processing time make it difficult to apply for routine identification. In contrast, MALDI-TOF MS offers rapid diagnosis and is commonly used in veterinary laboratories today. The major limiting factor for using this technique is the low environmental bacterial diversity in the commonly available databases. Here, we demonstrate that the sole use of the commercially available Bruker BioTyper database is not fully adequate for identifying *Vibrio* bacteria isolated from aquaculture farms. We therefore developed a new in-house database named Luvibase, composed of 23 reference MALDI-TOF mass spectra profiles obtained from *Vibrio* collection strains, mostly belonging to the Harveyi clade. The comparison of the accuracy of MALDI-TOF MS profiling and 16S rRNA gene sequencing revealed a lack of resolution for 16S rRNA gene sequencing. In contrast, MALDI-TOF MS profiling proved to be a more reliable tool for resolving species-level variations within the Harveyi clade. Finally, combining the Luvibase with the Bruker ver.9.0.0.0 database, led to successful identification of 47 *Vibrio* isolates obtained from moribund abalone, seabass and oysters. Thus, the use of Luvibase allow for increased confidence in identifying *Vibrio* species belonging to the Harveyi clade.

## Introduction

Bacteria belonging to the *Vibrio* genus, are highly abundant in aquatic environments (Thompson et al., 2004). Some of them are well-known enteric human pathogens such as *Vibrio parahaemolyticus*, *V. vulnificus*, or even *V. cholerae*, known as the causative agent of pandemic cholera (Bonnin-Jusserand et al., 2017). Other *Vibrio* species are pathogenic to aquatic animals such as several species belonging to the Harveyi clade. Associated with a great variety of crustaceans, mollusks, or fishes, these bacteria are particularly problematic in aquaculture, causing vibriosis outbreaks and high economic losses (Austin and Zhang, 2006; Novriadi, 2016; Ina-Salwany et al., 2019). The health risk to aquatic animals and the potential development of zoonosis heighten the need for rapid and reliable identification of *Vibrio* species belonging to the Harveyi clade (Cantas and Suer, 2014; Destoumieux-Garzón et al., 2018). Thus, in case of vibriosis outbreaks, appropriate control measures could be designed and rapidly applied.

The gold standard method for species identification is 16S rRNA gene sequencing (Chatterjee and Haldar, 2012). However, several studies have highlighted the poor discriminatory power of 16S rRNA sequence-based bacterial identification of *Vibrio* species, due to high genome similarity (Gomez-Gil et al., 2004; Hernandez and Olmos, 2004). Furthermore, this technique is often cost-ineffective and time-consuming, making it labor-intensive to apply in aquaculture facilities in a context of vibriosis outbreaks.

Thus, for rapid diagnosis, most veterinary laboratories routinely use matrix-assisted laser desorption ionization/time-of-flight mass spectrometry (MALDI TOF MS). The key benefits of this technique are the high throughput and speed combined with automation that provide rapid sample processing on a large scale (Fujinami et al., 2011; Singhal et al., 2015). Previous studies have demonstrated that MALDI-TOF MS is a valuable tool for discriminating among *Vibrio* spp. and closely related species (Hazen et al., 2009; Dieckmann et al., 2010; Eddabra et al., 2012; Schirmeister et al., 2014; Erler et al., 2015). However, the identification of bacteria relies on the comparison of experimental MALDI TOF MS profiles with reference MALDI TOF MS profiles recorded in a database. The most commonly and widely used database, is the commercially available Bruker BioTyper database (Bruker France S.A.S, Wissembourg, Germany). This latter is mainly intended for human clinical diagnosis. Although considerable efforts have recently been made to implement reference MALDI-TOF MS profiles from environmental strains, this database still lacks coverage of environmental bacterial isolates, particularly marine bacteria (Emami et al., 2012). Therefore, the use of this database for environmental surveys is limited.

To overcome this limitation, one suitable solution is to create an in-house database, adapted to the identification of the species of interest. For instance, Erler et al. (2015) created an available free in-house database, named VibrioBase, in order to improve the identification of *Vibrio* isolates at the species level. This database was mainly compiled using *Vibrio* environmental isolates potentially pathogenic for human and thus lacks spectra from potential aquatic animal pathogens. In particular, species belonging to the Harveyi clade, such as *V. owensii*, *V. rotiferianus*, *V. azureus*, or *V. jasicida* are missing from this database. Moreover, spectra from *V. harveyi* and *V. campbellii* have been included in a common *V. harveyi*/*campbellii* group, making their discrimination impossible. Previous studies have already highlighted the need to find accurate tools allowing discrimination between these two species (Gomez-Gil et al., 2004; Ruwandeepika et al., 2010; Cano-Gomez et al., 2011). The impact of *V. campbellii* in aquaculture facilities may indeed have been underestimated due to unreliable identification.

The aim of this study was to improve the identification of *Vibrio* belonging to the Harveyi clade to the species level using MALDI-TOF MS profiling. More specifically, our objective was to develop a method for the accurate identification of pathogens obtained from moribund abalone (*Haliotis tuberculata*), seabass (*Dicentrarchus labrax*), and oyster (*Crassostrea gigas*) from aquaculture farms. These strains have been previously identified using PCR and sequencing by Mougin et al. (2020). Given that the MALDI BioTyper was not adequate for species identification, we constructed an in-house database, named Luvibase. Reference MALDI-TOF mass spectra profiles, also shortened as mass spectra profiles (MSPs), obtained from 23 *Vibrio* collection strains were incorporated into the Luvibase. We then compared MALDI-TOF MS profiling with 16S rRNA gene sequencing for species identification. Finally, the identification of 62 environmental isolates was performed using the Bruker BioTyper ver.9.0.0.0 database supplemented with the Luvibase, to provide evidence of the usefulness of this augmented database.

## Materials and Methods

### Bacterial Strains and Culture Conditions

A total of 85 strains were used in this study (Table 1). These strains were described and identified by PCR, sequencing of house-keeping genes or whole genome sequencing in a previous study (Mougin et al., 2020). Briefly, (i) 38 collection strains were obtained from the Belgian Coordinated Collections of Microorganisms (BCCM/LMG), the German Collection of Microorganisms and Cell Cultures (DSMZ), the Spanish Type Culture Collection (CECT), and the Institut Pasteur Collection (CIP), (ii) 35 isolates were obtained from a fish farm (Aquanord-Ichtus, Gravelines, France) raising seabass *Dicentrarchus labrax* and (iii) 12 isolates were isolated by the French Research Institute for the Exploitation of the Sea (IFREMER) from oysters (*Crassostrea gigas*) and abalone (*Haliotis tuberculata*).

**TABLE 1:**
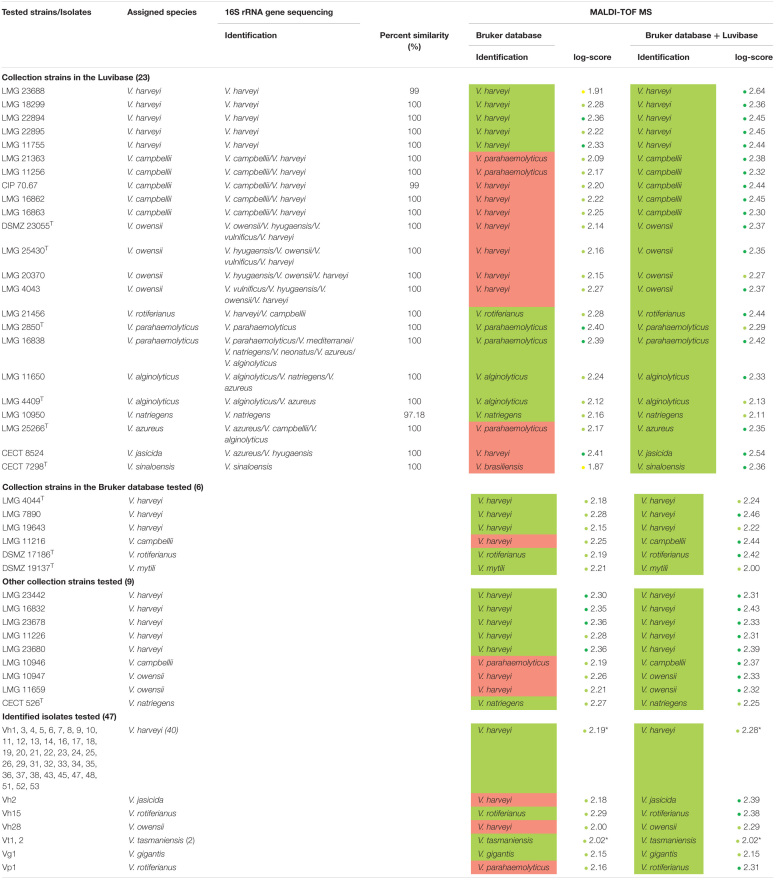
Comparison between 16S rRNA gene sequencing identification results and MALDI-TOF MS profile-based identification of *Vibrio* species using the Bruker BioTyper ver.9.0.0.0 database alone and supplemented with the Luvibase.

Bacterial isolates were previously identified using PCR and sequencing by Mougin et al. (2020). Green shading, MALDI-TOF MS identifications matching the assigned species identifications; red shading, MALDI-TOF MS identifications not matching the assigned species identifications. *Mean of log-scores. A log-score (i) greater than 2.3 indicates a highly probable identification at the species level (dark green circle), (ii) between 2.0 and 2.3 indicates a probable identification at the species level (light green circle), (iii) between 1.7 and 2.0 indicates a probable identification at the genus level only (yellow circle), and (iv) below 1.7 indicates no significant similarities between the reference MSPs included in the databases and the experimental MALDI-TOF MS profiles.

Before all experiments, bacteria were first sub-cultured on marine agar (MA) (Difco Laboratories, Detroit, MI, United States), and incubated for 24 h, at growth temperature. Second, a single colony forming unit (CFU) was plated on MA (Difco Laboratories) and incubated 24 h, at growth temperature, to ensure the purity of the isolates. This growth temperature was 37°C, except for LMG 11216^T^, Vh2, Vh3, Vt1, Vt2 and Vg1, for which the growth temperature was 25°C.

### 16S rRNA Gene Sequencing and Sequence Analysis

The DNA of 23 *Vibrio* collection strains was extracted from pure culture, isolated from MA agar using the DNeasy Blood & Tissue kit (Qiagen, Hilden, Germany) and an automated nucleic acid extractor QIAcube Connect (Qiagen), according to the manufacturer’s instructions. The final elution volume was 100 μL. The extracted DNA was then stored at −20°C until use.

PCR amplification of the 1,465 bp 16S rRNA gene fragment (V1-V8 region) was performed as described in Bauer et al. (2018) using the primers 27f (5′-AGAGTTTGATCMTGGCTCAG-3′) and 1492r (5′- ACGGYTACCTTGTTACGACTT-3′). The primers were synthetized by TIB MOLBIOL (TIB MOLBIOL Syntheselabor GmbH, Berlin, Germany) and were suspended in nuclease-free water to reach a final concentration of 10 μM and stored at −20°C. The PCR reaction mixture contained 25 μL of 2 × Platinum^TM^ Green Hot Start PCR Master Mix (Invitrogen, Carlsbad, CA, United States), 1 μL of each primer (10 μM), 5 μL of template DNA, and 18 μL of nuclease-free water to a final volume of 50 μL. The PCR reaction was run on a Thermal Cycler (Applied Biosystems, Forster City, CA, United States), under the following conditions: 3 min at 95°C, followed by 30 cycles of 30 s at 95°C, 30 s at 55°C, and 1 min at 72°C. The final cycle was followed by an additional 7 min of extension at 72°C. Each run included a no template control (NTC), DNA-free. The size of the PCR product was verified using ethidium bromide agarose gel electrophoresis (2%). The PCR products of expected size were then paired-end sequenced, using Sanger sequencing by GenoScreen (Lille, France).

Sequence analysis was performed using the nucleotide BLAST search program with the GenBank database (NCBI). A strict identification criterion of ≥99% sequence identity for identification to the species level was applied (Janda and Abbott, 2007). A distance tree was generated using CLC Genomics Workbench ver.20.0.2. The unweighted pair grouping method with arithmetic-mean (UPGMA) hierarchical clustering algorithm was used with the Jukes-Cantor distance.

### Preparation of Bacterial Extracts and Target-Loading for MALDI-TOF MS Profiling

The cellular proteins of 23 *Vibrio* collection strains were extracted from bacterial cells according to manufacturer’s recommendations (Bruker France S.A.S., Wissembourg, Germany). Fresh colonies were picked with a 1 μL inoculation loop and placed in 300 μL of water (ULC/MS-CC/SFC, Biosolve Chimie, Dieuze, France). A volume of 900 μL of pure ethanol (VWR International, Fontenay-sous-Bois, France) was added. The tube was vortexed for 1 min and centrifuged at 13,000 × *g* for 2 min. The supernatant was removed and the pellet was centrifuged a second time for 15 s, to eliminate as much ethanol as possible. Thereafter, pellets were air dried under a fume hood for 20 min. Once dried, pellets were dissolved into 30 μL of 70% formic acid (Alfa Aesar, Kandel, Germany). An equal volume of 100% acetonitrile (Biosolve Chimie) was added and the solution was mixed carefully by pipetting up and down. The tube was then centrifuged at 13,000 × *g* for 2 min. The extracts were stocked at −20°C until use. Subsequently, 1 μL of the supernatant from each protein extract was spotted onto a clean ground steel MTP 384-target plate (Bruker France S.A.S.) in 24 replicates. After air-drying, each spot was overlaid with 1 μL of a 10 mg.mL^–1^ matrix solution of α-cyano-4-hydroxycinnamic acid (CHCA) in 50% acetonitrile and 2.5% trifluoroacetic acid (Sigma-Aldrich, St. Louis, MO, United States).

### Acquisition of MALDI-TOF MS Profiles

MALDI-TOF-MS analysis was performed using an Autoflex SpeedTM (Bruker France S.A.S.), running Flexcontrol 3.4 software (Bruker France S.A.S.). The Bruker bacterial test standard (BTS, Bruker France S.A.S.) was used to calibrate the mass spectrometer before each run, according to the manufacturer’s recommendations. Mass spectra were acquired in the positive linear ion mode across a mass-to-charge (*m/z*) ratio of 2,000–20,000 Da. The mass spectra were acquired using the manufacturer’s automatic method MBT_FC.par. For each MALDI-TOF MS profile, mass spectra obtained from 5,000 laser shots in 1,000 shot steps performed randomly on different areas of the spot were accumulated.

### Creation of the Luvibase, an In-House Main Spectral Profile Database

Each of the 23 MALDI-TOF MS profiles obtained from 24 MALDI-target spots were analyzed and processed using FlexAnalysis ver.3.4 (Bruker France S.A.S.). MALDI-TOF MS profiles with a signal intensity lower than 10^4^ arbitrary units (a.u.) were discarded. A minimum of 18 MALDI-TOF MS profiles were used to create an MSP using MALDI BioTyper Compass Explorer ver.4.1 software (Bruker France S.A.S.). All 23 MSPs were registered in an in-house database called Luvibase. In order to ensure the repeatability of the assay, tree cultures of each strain were performed and the similarity of mass spectra of each culture was confirmed.

An MSP dendrogram was generated using MALDI BioTyper Compass Explorer v4.1 (Bruker France S.A.S.) using the Euclidean distance measure and a complete coverage algorithm.

### Bacterial Identification Using MALDI-TOF MS Profiling

A single CFU, isolated from a fresh bacterial culture, was spotted onto a clean ground steel MTP 384-target plate (Bruker France S.A.S.) in four replicates. Then, 1 μL of 70% formic acid (Alfa Aesar) was overlaid on each spot. After air-drying at room temperature, 1 μL of CHCA solution in 50% acetonitrile and 2.5% trifluoroacetic acid (Sigma-Aldrich) was added to each spot and air-dried. Subsequently, the above mentioned MALDI-TOF parameters were used to acquire the MALDI-TOF MS profiles.

Each MALDI-TOF MS profile was first compared to the MSPs of the Bruker BioTyper database ver.9.0.0.0 (Bruker France S.A.S.) and then to the MSPs of the Bruker BioTyper database ver.9.0.0.0 supplemented with the Luvibase. The concordance degree between experimental MALDI-TOF MS profiles and the MSPs was evaluated using the log-score calculated by MALDI BioTyper Compass Explorer v4.1 (Bruker France S.A.S.). A log-score greater than 2.3 indicates a highly probable identification at the species level. A log-score between 2.0 and 2.3 indicates a probable identification at the species level. A log-score between 1.7 and 2.0 indicates a probable identification at the genus level only. A log-score below 1.7 indicates no significant similarities between MSPs included in the databases and the tested profile.

## Results

### Identification of *Vibrio* Bacteria Using the Bruker Ver.9.0.0.0 Database

We acquired MALDI-TOF MS profiles for 85 *Vibrio* bacterial strains, classified into 13 *Vibrio* species mostly belonging to the Harveyi clade, were acquired. These spectra were first compared to the MSPs included in the Bruker ver.9.0.0.0 database. The bacterial identifications previously obtained in the study of Mougin et al. (2020) were then compared with the MALDI-TOF MS profile-based bacterial identifications. The matching rates were 100% at the genus level and only 75.3% at the species level, assuming a log-score of at least 2.0 required for probable species identification (Table 1). A total of 19 bacterial strains were misidentified using the Bruker ver.9.0.0.0 database, involving the six following species: *V. campbellii*, *V. owensii*, *V. azureus*, *V. jasicida*, *V. sinaloensis*, and *V. rotiferianus*. (i) All *V. campbellii* collection strains, LMG 21363, LMG 11256, CIP 70.67, LMG 16862, LMG 16863 LMG 11216, and LMG 10946 were misidentified as *V. parahaemolyticus* or *V. harveyi* (log-score > 2); (ii) all *V. owensii* collections strains, DSMZ 23055^T^, LMG 25430^T^, LMG 20370, LMG 4043 LMG 11659, LMG 10947 and the *V. owensii* isolate Vh28 were misidentified as *V. harveyi* (log-score > 2); (iii) the *V. jasicida* collection strain CECT 8524 and the *V. jasicida* isolate Vh2 were misidentified as *V. harveyi* (log-score > 2); (iv) the *V. azureus* collection strains LMG 25266^T^ was misidentified as *V. parahaemolyticus* (log-score > 2); (v) the *V. rotiferianus* isolate Vp1 was misidentified as *V. parahaemolyticus* whereas the *V. rotiferianus* collection strain LMG 21456 was correctly identified (log-score > 2) and (vi) the *V. sinaloensis* collection strain CECT 7298^T^ was identified to the genus level only (1.7 < log-score < 2). Therefore, we constructed an in-house database to provide a reliable identification of bacteria belonging to these six species.

### Creation of the In-House Database, Luvibase

**FIGURE 1:**
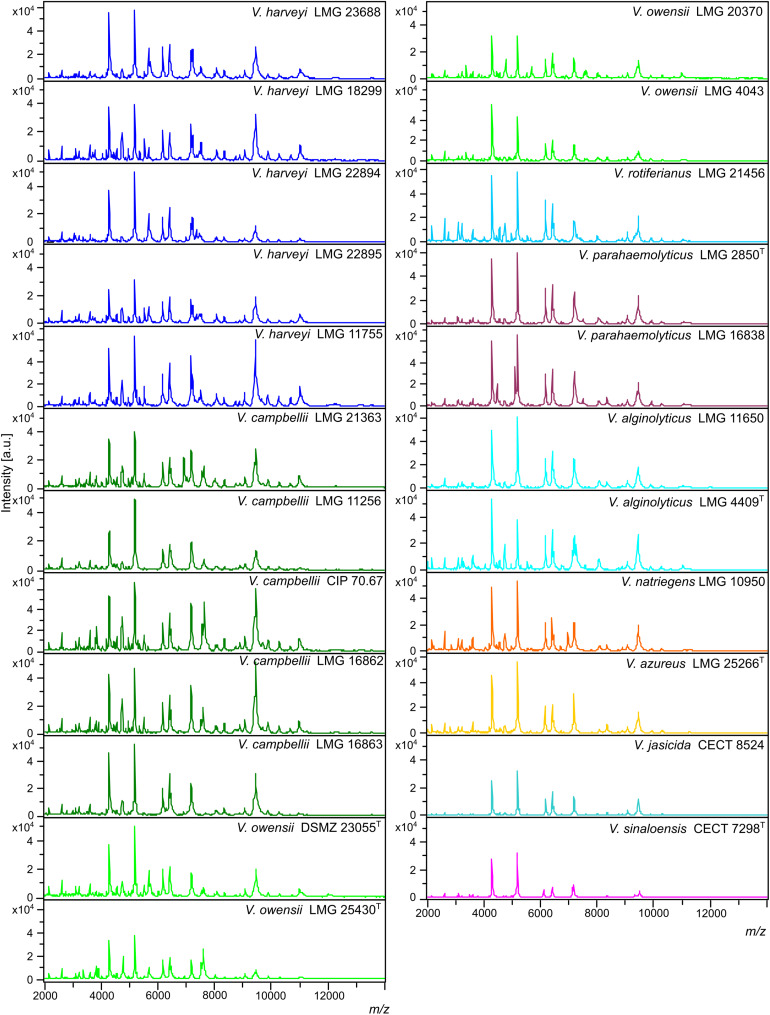
Main spectral profiles (MSPs) of the 23 *Vibrio* strains included in the Luvibase. The whole 2,000–14,000 *m/z* spectra are shown (a.u., arbitrary units; *m/z*, mass to charge ratio).

MSPs, derived from 23 *Vibrio* collection strains, were created and recorded in the in-house database: Luvibase (Tables 1, 2). The Luvibase includes the MSPs of 12 collection strains classified into the six species that yielded unreliable identifications: *V. campbellii*, *V. owensii*, *V. azureus*, *V. jasicida*, *V. sinaloensis*, and *V. rotiferianus*. In addition, the MSPs of 11 collection strains classified as *V. harveyi*, *V. parahaemolyticus*, *V. alginolyticus*, and *V. natriegens* were included in the Luvibase. As illustrated in Figure 1, each MSP of the 10 *Vibrio* species implemented in the Luvibase, gave a unique and reproducible species-specific MSP. Minor visual differences were perceptible between these MSPs: they were clearly similar but not identical.

**TABLE 2:** Number of *Vibrio* strains included in the Bruker BioTyper database ver.9.0.0.0 and in the Luvibase.

Species	No. of strains in the Bruker database	No. of strains in the Luvibase
*Vibrio harveyi*	7	5
*Vibrio campbellii*	1	5
*Vibrio owensii*	0	4
*Vibrio alginolyticus*	5	2
*Vibrio rotiferianus*	1	1
*Vibrio parahaemolyticus*	9	2
*Vibrio natriegens*	2	1
*Vibrio azureus*	0	1
*Vibrio jacicida*	0	1
*Vibrio sinaloensis*	0	1

### Identification of the Strains Included in the Luvibase Using 16S rRNA Gene Sequencing

The 16S rRNA gene sequences (V1-V8 region) of the 23 strains included in the Luvibase were obtained and identification was performed using the nucleotide BLAST search program with the GenBank database (NCBI). For most of the fragments, the sequence similarity obtained was above the strict threshold defined for identification at the species level (≥99% sequence similarity). The sequence similarity obtained from fragment acquired from the LMG 10950 *V. natriegens* collection strain was below the threshold (97.48%), and was thus identified at the genus level only. Nevertheless, 15/22 strains could not be identified due to high sequence similarities with more than one species (Table 1): strains belonging to *V. campbellii*, *V. owensii*, *V. rotiferianus*, *V. alginolyticus*, *V. azureus*, and *V. jasicida* could not be correctly identified. On both *V. parahaemolyticus* strains, only the LMG 2850^T^ collection strain was correctly identified whereas the LMG 16838 collection strain was incorrectly identified. Strains belonging to *V. harveyi* and *V. sinaloensis* were correctly identified.

### Comparison of 16S rRNA Gene Sequencing to MALDI-TOF Profiling for Identification of Species Included in the Luvibase

**FIGURE 2:**
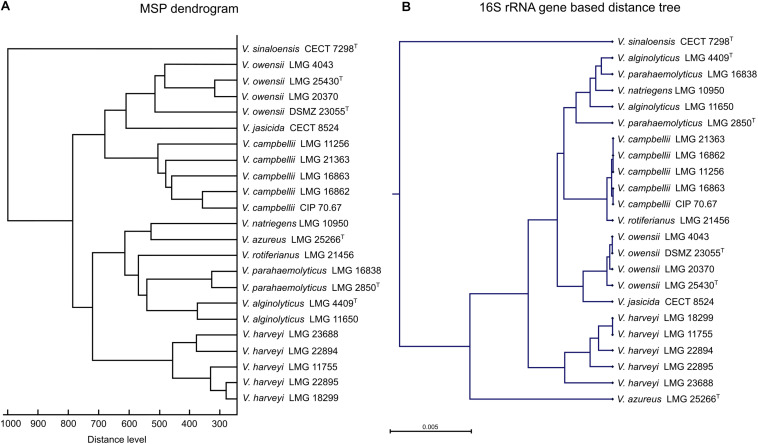
Comparison of **(A)** main spectral profile (MSP) dendrogram based on MALDI-TOF MS profiling analysis and **(B)** 16S rRNA gene-based distance tree resulting based on 16S rRNA gene sequencing of 23 *Vibrio* strains included in the Luvibase.

To evaluate the agreement between species discrimination using 16S rRNA gene sequencing and MALDI-TOF MS profiling, a hierarchical distance tree, based on the 16S rRNA gene sequences obtained from the 23 strains in the Luvibase, and a MSP dendrogram based on the MSPs included in the Luvibase were generated (Figure 2).

Using both techniques, all strains belonging to the Harveyi clade were grouped together and *V. sinaloensis*, which belongs to the Orientalis clade, was easily distinguishable. Nevertheless, comparison between the MSP dendrogram and the 16S rRNA gene-based distance tree revealed that the clusters within the Harveyi clade differed between the two techniques. Analysis of clusters resulting from MALDI-TOF MS profiling highlighted species-specific groups, but analysis of clusters from 16S rRNA gene sequencing were not all species-specific. The 16S rRNA gene sequences of *V. alginolyticus*, *V. natriegens*, and *V. parahaemolyticus* clustered together. These findings suggest that the two techniques have different taxonomic resolution.

### Identification of *Vibrio* Bacteria Using the Bruker Ver.9.0.0.0 Database Supplemented With the Luvibase

To evaluate the effectiveness of the Luvibase for identification of unknown isolates, the 85 *Vibrio* bacteria were re-identified. The Bruker BioTyper database supplemented with the Luvibase and bacterial identification results are summarized in Table 1. The matching rates were 100% at both the genus and the species levels, assuming a log-score of at least 2.0 required for probable species identification (Table 1).

Overall, the mean of log-score obtained with the Bruker BioTyper ver.9.0.0.0 database was 2.21 ± 0.12, whereas the mean of log-score obtained with the Bruker BioTyper ver.9.0.0.0 database supplemented with the Luvibase was 2.34 ± 0.12. Thus, the Bruker BioTyper ver.9.0.0.0 database gave mostly “probable species identifications” (log-score < 2.3) while the augmented database gave “secure species identifications” (log-score ≥ 2.3).

All experiments were validated by negative and positive control spots. The negative control spots yielded no peaks or faint profiles that could not be identified by the system, and the positive control spots yielded the expected *Escherichia coli* DH5alpha identification score of 2.0–2.5.

## Discussion

Genomic-based methods for *Vibrio* identification, such as PCR and sequencing, offer accurate identification, assuming the use of a specific and reliable target gene (Cano-Gomez et al., 2009). However, they are also labor-intensive, requiring highly trained operators and are not particularly suitable for proceeding a large quantity of samples. On the contrary, MALDI-TOF MS is an automated, rapid, cost-effective and useful tool for bacterial species identification. This technique has been successfully used to discriminate closely related *Vibrio* species (Dieckmann et al., 2010; Erler et al., 2015). The major limitation is the lack of MSP diversity in the main available spectral profile databases (Clark et al., 2013).

The Bruker BioTyper ver.9.0.0.0 database includes 111 MSPs obtained from 53 different *Vibrio* species (Supplementary Table S1). As expected, strains belonging to *V. owensii, V. azureus, V. jasicida* and *V. sinaloensis* species were not correctly identified because their MSPs are not included. Consistently, the *V. sinaloensis* spectrum did not specifically match any MSP and was identified to the genus level only. However, the MALDI-TOF MS profiles for *V. owensii*, *V. azureus* and *V. jasicida* matched the MSPs of other *Vibrio* strains with log scores ≥2 or ≥2.3, the recommended standard thresholds for probable or secure species identification, respectively. These inaccurate bacterial identification matches highlight the high similarity of MALDI-TOF MS profiles between *Vibrio* species. In contrast, the Bruker BioTyper ver.9.0.0.0 database does include one MSP for *V. campbellii* and *V. rotiferianus*. All MALDI-TOF MS profiles from strains classified as *V. campbellii* matched the MSPs of other *Vibrio* species. The MALDI-TOF MS profile from a *V. rotiferianus* isolate did not match the MSP of its own species. Nonetheless, the MALDI-TOF MS profiles from the other *V. rotiferianus* strains correctly matched the species MSP. These findings suggest that including only one MSP in a MSP database is not sufficient for secure species identification, perhaps partly due to the high heterogeneity between environmental isolates. For instance, the geographic diversity of *V. parahaemolyticus* isolates can lead to divergences in MALDI-TOF MS profiles (Hazen et al., 2009). To ensure high epidemiological coverage, more MSPs must be recorded in the bacterial identification databases. Thus, the reliable identification of the causative agents of disease outbreaks in aquaculture requires the construction of a database including different strains of *V. parahaemolyticus* and related species.

Here, we constructed a new in-house database, named Luvibase, in order to improve the identification of *Vibrio* belonging to the Harveyi clade at the species level. The MSPs of 23 *Vibrio* species were acquired and included. Overall, the use of the Luvibase in addition to the Bruker BioTyper ver.9.0.0.0 database provided more reliable identification than the sole use of the Bruker BioTyper ver.9.0.0.0 database. Moreover, the cluster analysis highlighted the capacity of MALDI-TOF MS profiling to discriminate between closely related species. Remarkably, the method allowed accurate identification and discrimination between *V. harveyi*, *V. campbellii* and *V. rotiferianus* whose 16S rRNA gene sequences have more than 99% similarity (Gomez-Gil et al., 2003, 2004).

16S rRNA gene sequencing is currently considered the gold standard for bacterial identification. However, in our study, only a few strains could be correctly identified to the species level due to high 16S rRNA gene sequence similarities between *Vibrio* species belonging to the Harveyi clade. Moreover, the 16S rRNA gene-based distance tree analysis highlighted a close relationship between the 16S rRNA gene sequences of *V. alginolyticus*, *V. natriegens*, and *V. parahaemolyticus*. Importantly, the discrimination between these species proved to be laborious, because they shared high similarity in their 16S rRNA gene sequences (Dorsch et al., 1992; Thompson et al., 2009). Instead of the 16S rRNA gene sequencing, Dieckmann et al. (2010) demonstrated that the partial *rpoB* gene sequencing can resolve species-level variations between *V. alginolyticus* and *V. parahaemolyticus*. Thus, the 16S rRNA gene is not a reliable target gene for discrimination between these species. Generally speaking, 16S rRNA gene sequencing is not suitable for species identification within the Harveyi clade which form a tight cluster with more than 99% sequence similarities (Cano-Gomez et al., 2011). In contrast, MALDI-TOF MS profiling had higher resolving power. This technique is based on the analysis of a large spectrum of peptides/proteins generated from the whole cell, while 16S rRNA gene sequencing is based only on a 1,500 bp gene. Thus, MALDI-TOF MS profiling enables the characterization of proteomic differences between bacteria with highly similar 16S rRNA gene sequences. For this reason, in our study, MALDI-TOF MS profiling gave a more accurate bacterial identification than 16S rRNA gene sequencing. In addition, there is no universal consensus of an established threshold value for sequence similarity above which strains can unambiguously be identified to the species level, using 16S rRNA gene sequencing (Janda and Abbott, 2007). Attempts to propose an optimal threshold of 98.65% when using 16S rRNA gene sequencing for species identification are stymied by the fact that some species share more than 99% gene sequence similarity (Kim et al., 2014). In contrast, the log-score threshold values for MALDI-TOF MS identification are well-defined and universally applicable. Overall, the implementation of the Bruker BioTyper database along with the Luvibase, for bacterial identification using MALDI-TOF MS appears to be an appropriate, convenient, and powerful tool to discriminate between closely related species belonging to the Harveyi clade.

Standard conditions must be well-defined and applied to ensure reliability and reproducibility of MALDI-TOF MS identification. The divergence between methods can lead to divergence between mass signals and thus MALDI-TOF MS profiles (Santos et al., 2016). Several studies have highlighted the importance of cell growth conditions, culture age, matrices, solvents and method of sample application on the target plate (Evason et al., 2001; Williams et al., 2003; Vargha et al., 2006). These parameters may affect the spectra and, thus the associated identifications. However, there is a controversy regarding the impact of growth conditions. Some studies claimed that even if the spectrum is slightly modified, identification to the species level remain possible (Valentine et al., 2005; Usbeck et al., 2013). For instance, the use of different culture media for *Yersinia enterocolitica*, *Bacillus subtilis*, and *Escherichia coli* identification by MALDI-TOF MS profiling showed an unambiguous core set of proteins, ensuring reliable identification regardless the culture media (Valentine et al., 2005). Recently, the identification of *V. anguillarum* pathogens isolated from seabass and seabream (*Sparus aurata*) revealed that differences in culture media and incubation period have no effect on MALDI-TOF MS profiles but divergences in growth temperature may affect MALDI-TOF MS profiles (Kazazić et al., 2019). Importantly, because the spectra of different species may not be affected by the same parameters, the effect of altering procedures should be studied for each single species. To our knowledge, no such studies have been undertaken for *Vibrio* species belonging to the Harveyi clade. Procedures clearly need to be standardized to ensure consistent identification between laboratories.

## Conclusion

This work revealed discrepancies between *Vibrio* species discrimination using 16S rRNA gene sequencing and MALDI-TOF MS profiling. In our study, MALDI-TOF MS profiling provided better resolution because this technique is based on the analysis of a large spectrum of peptides/proteins from the whole cell, whereas 16S rRNA gene sequencing is based on a single 1,500 bp gene. Therefore, 16S rRNA gene sequences exhibit high similarities between *Vibrio* species. However, the success of bacterial identification using MALDI-TOF profiling relies on the reference strains registered in databases. As demonstrated in the present study, the sole use of the Bruker BioTyper ver.9.0.0.0 database is not suitable for identification of *Vibrio* isolates from aquaculture farms. For that reason, we constructed the Luvibase, an in-house database suitable for the discrimination of *Vibrio* species belonging to the Harveyi clade. The Luvibase combined with the Bruker BioTyper database can be used for pathogen identification in aquaculture farms. The combination of these both databases led to reliable identification of the strains belonging to *V. campbellii*, *V. owensii*, *V. azureus*, *V. jasicida*, *V. sinaloensis*, and *V. rotiferianus*, whereas the use of the Bruker BioTyper database alone gave incorrect identifications. Therefore, veterinary laboratories can use the augmented database for the rapid and reliable diagnosis of vibriosis outbreaks. In the case of an identified outbreak, suitable control methods could be then undertaken to limit the spread of the disease. The inclusion of additional environmental isolates in the Luvibase could then expand the coverage and the use of this technique.

## Data Availability Statement

The datasets generated for this study can be found in the NCBI BioProject ID: PRJNA663549, https://www.ncbi.nlm.nih.gov/bioproject/663549.

## Author Contributions

JM: methodology, formal analysis, investigation, writing original draft, and visualization. CF: methodology, validation, and writing and reviewing. RR: investigation, formal analysis, and writing and reviewing. MB-J: conceptualization, validation, writing and reviewing, and supervision. TG: validation, writing and reviewing, supervision, project administration, and funding acquisition. CLB: conceptualization, validation, investigation, writing and reviewing, supervision, and project administration. All authors contributed to the article and approved the submitted version.

## Conflict of Interest

The authors declare that the research was conducted in the absence of any commercial or financial relationships that could be construed as a potential conflict of interest.
