# Supplementary material for: Rapid Identification of Vibrio Species of the Harveyi Clade Using MALDI-TOF MS Profiling With Main Spectral Profile Database Implemented With an In-House Database: Luvibase
Source: Front Microbiol. 2020 Oct 9;11:586536. doi: 10.3389/fmicb.2020.586536 (PMC7581793; doi:10.3389/fmicb.2020.586536)
Supplement: Supplementary file 1 [file Table_1.DOCX]

**Supplementary Table 1.** Species list and number of *Vibrio* strains included in the Bruker BioTyper database ver.9.0.0.0.

| Species | No. of strains in the Bruker database |
| --- | --- |
| *Vibrio aerogenes* | 1 |
| *Vibrio aestuarianus* | 4 |
| *Vibrio agarivorans* | 1 |
| *Vibrio albensis* | 1 |
| *Vibrio alginolyticus* | 5 |
| *Vibrio anguillarum* | 7 |
| *Vibrio brasiliensis* | 1 |
| *Vibrio campbellii* | 1 |
| *Vibrio chagasii* | 1 |
| *Vibrio cincinnatiensis* | 1 |
| *Vibrio coralliilyticus* | 1 |
| *Vibrio cyclitrophicus* | 1 |
| *Vibrio diazotrophicus* | 6 |
| *Vibrio ezurae* | 1 |
| *Vibrio fluvialis* | 3 |
| *Vibrio fortis* | 1 |
| *Vibrio furnissii* | 3 |
| *Vibrio gazogenes* | 1 |
| *Vibrio gigantis* | 1 |
| *Vibrio harveyi* | 7 |
| *Vibrio hispanicus* | 1 |
| *Vibrio ichthyoenteri* | 2 |
| *Vibrio kanaloae* | 1 |
| *Vibrio lentus* | 1 |
| *Vibrio mediterranei* | 2 |
| *Vibrio metschnikovii* | 2 |
| *Vibrio mimicus* | 1 |
| *Vibrio mytili* | 1 |
| *Vibrio natriegens* | 2 |
| *Vibrio navarrensis* | 5 |
| *Vibrio neptunius* | 1 |
| *Vibrio nereis* | 1 |
| *Vibrio nigripulchritudo* | 1 |
| *Vibrio ordalii* | 1 |
| *Vibrio orientalis* | 1 |
| *Vibrio ostreicida* | 3 |
| *Vibrio pacinii* | 1 |
| *Vibrio parahaemolyticus* | 9 |
| *Vibrio pectenicida* | 2 |
| *Vibrio pelagius* | 1 |
| *Vibrio penaeicida* | 1 |
| *Vibrio pomeroyi* | 1 |
| *Vibrio ponticus* | 1 |
| *Vibrio proteolyticus* | 1 |
| *Vibrio rotiferianus* | 1 |
| *Vibrio ruber* | 1 |
| *Vibrio rumoiensis* | 1 |
| *Vibrio scophthalmi* | 2 |
| *Vibrio splendidus* | 1 |
| *Vibrio superstes* | 1 |
| *Vibrio tasmaniensis* | 1 |
| *Vibrio vulnificus* | 11 |
| *Vibrio xuii* | 1 |
| **TOTAL** | 111 |
